# Evidence of Validity and Normative Values of a New Auditory Backward Masking Test

**DOI:** 10.3390/jcm11174933

**Published:** 2022-08-23

**Authors:** Renata Filippini, Carlos Alberto Leite Filho, Gabriela Melo Santos Bonassa Barros, Frank E. Musiek, Eliane Schochat

**Affiliations:** 1 Department of Physical Therapy, Speech-Language Pathology and Occupational Therapy, School of Medicine, University of São Paulo, São Paulo 05360-160, Brazil; 2 Department of Speech, Language, and Hearing Sciences, The University of Arizona, Tucson, AZ 85721-0071, USA

**Keywords:** central auditory processing, auditory temporal processing, backward masking, auditory perception, central auditory processing disorder, hearing tests

## Abstract

There are still no valid, clinically feasible instruments to assess backward masking (BM), an auditory temporal processing (ATP) phenomenon. The aim of this study was to develop, standardize and present evidence of validity for a behavioral test for BM assessment. Young adults were submitted to a BM test (BMT), where they were asked to identify a 1000 Hz pure tone followed by a narrowband noise with interstimulus intervals of 0 to 400 ms and signal-to-noise ratio (SNR) between −20 and −30 dB. The correct response rate and target sound detection threshold were calculated, and the results compared with those of young adults with abnormal ATP tests and older adults. Diagnostic accuracy analyses were carried out. Young adults with normal ATP obtained an average correct response rate of 89 and 87% for SNR −20 and −30 dB, respectively, with average thresholds between 10 and 15 ms and no difference between the left and right ears. Results were more consistent at SNR −20 dB, and the best diagnostic accuracy was obtained for SNR −20 dB, with good specificity, but low sensitivity. Normative values were obtained for the BMT, which proved to be clinically feasible, with preliminary evidence of validity.

## 1. Introduction

Time is one of the most important variables that underlies the perception and understanding of auditory information. Music and speech, for example, are made up of linked sounds whose characteristics, such as order, pitch, duration and intensity, allow the sounds to be recognized. These characteristics vary over short time intervals, requiring excellent auditory temporal processing (ATP) in order to be understood [1].

ATP is defined as the perception of a sound or a change in sound within a limited or defined time frame [2,3] and can be divided into four categories: (1) temporal ordering; (2) temporal resolution; (3) temporal integration; and (4) temporal masking [4,5].

Theories have been developed that correlate ATP alterations with speech, language and writing disorders [6,7,8,9], largely involving temporal ordering [10,11,12] and masking [13,14,15,16]. However, there are discrepancies between different authors and studies regarding these relationships [17,18]. Authors who oppose these theories point to the considerable variability of the results obtained, different methodologies [19], and the influence of cognitive processes [20,21,22]. As such, there is a need to develop tools to assess ATP, with results that can be easily replicated in different clinical and research scenarios in order to standardize the data obtained and improve diagnostic reliability.

Although there are well-documented clinical measures available to evaluate temporal ordering and resolution, there are no feasible standardized clinical tests for temporal integration and masking [5]. The present study aimed to develop a test to assess temporal masking. This category was chosen because, in addition to its previously mentioned relationship with language disorders, it has been investigated since the 1950s with no consensus on the mechanisms involved in this phenomenon [23].

Temporal masking is a phenomenon whereby the detection threshold of a sound stimulus is obscured by a masking sound (masker) presented before (forward masking) or after the stimulus (backward masking) [2,5]. Backward masking (BM) seems to be closely related to language disorders [13,14,24,25,26] because it would influence perception of the two stimuli presented in sequence, as in the case of coarticulation [4]. Additionally, evidence on the low involvement of peripheral processes [27], late maturation [28,29] and age-related difficulties [30] indicates that BM is more involved in central auditory function than forward masking. 

A pilot study [31] demonstrated the feasibility and applicability of a clinical BM test (BMT) that analyzes the variation in interstimulus intervals (ISIs) rather than intensity, which is the focus of traditional psychoacoustic studies. The silent gaps between stimuli have been shown to be highly influential in BM and are inversely proportional to masking efficiency [4]; however, high-intensity maskers do not necessarily cause greater masking [32].

As such, this study presents a new version of the BMT aimed at assessing the effect of temporal masking. First, the test was applied to a population of adults with no auditory or cognitive disorders (normal adults—NA) in order to determine normative values. Next, the test was also applied to a population with abnormal auditory temporal processing tests (abnormal adults—AA) and older adults (OA) with no cognitive impairment, with a view to verifying the diagnostic accuracy of the proposed normative values. The AA group was chosen to test the cutoff points due to the shared behavioral, neurophysiological, and neuroanatomical correlates of the ATP tests [1,2,33], while the OA group was expected to perform worse at the BMT test due to a decline in general perceptual processing [34,35]. 

## 2. Materials & Methods

### 2.1. The BMT

The BMT was developed based on established information regarding temporal masking [4,32,36,37,38,39,40]. The test involves presenting a 20 ms long (10 ms onset/offset) pure tone (1000 Hz) and a 200 ms long narrowband masking noise (600 Hz to 1400 Hz), separated by one of eight possible intervals: 400, 200, 100, 50, 30, 20 10 and 0 ms. 

The test was produced digitally (WAV* format) using Sound Forge Pro 10.0^®^ software (Sony Creative Software Inc., Middleton, WI, USA), and the tracks were recorded at a rate of 44,100 Hz and 16-bit resolution. The two stimuli (target sound and masker) were recorded on different channels so that each stimulus could be presented at different intensities, or the test could be performed in a monotic (both stimuli applied to the same ear) or dichotic format (one in each ear). 

**Figure 1:**
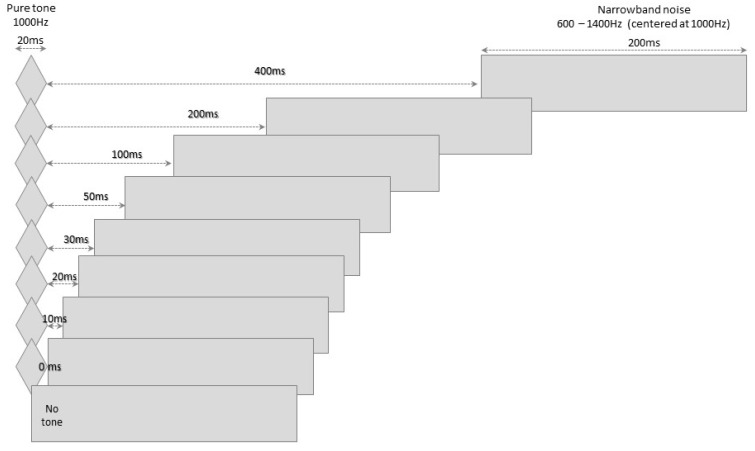
Representation of the stimuli and intervals of the backward masking test.

Two test tracks and one training track were recorded. The training track consisted of 15 items (i.e., presentation of a pure tone followed by a masker), for which only 20 to 400 ms ISIs were used. In the test tracks, each of the eight ISIs was presented 6 times, totaling 48 items. The tracks also contained another 12 items in which only the masker was presented (i.e., sham item), in order to control the reliability of the responses (Figure 1). The 60 items of the test tracks were randomly distributed, but in cycles that prioritized balanced distribution of the ISIs throughout the tracks. The items were preceded by narrations indicating the order of presentation/item number and presented at ~2.5-s intervals for an application time of around 5 min for each track. 

Test application involved monotic presentation (pure tone and masker in the same ear) in both ears. The masker was presented at a fixed intensity of 40 dBSL (re: 1000 Hz threshold for each individual) and the tone was presented at an SNR of −20 and −30 dB. These ratios were chosen because they exhibited the lowest variability and smallest ceiling effect in the pilot study [31]. 

Participants were asked to press a button whenever they heard the pure tone. Responses were marked on a specific answer sheet (Annex S1), and the percentages of total correct responses for each ISI were calculated at the end. The results of the BMT were the percentage of correct responses (BMTP) and the shortest ISI in which the participant heard the pure tone most of the time (4/6 correct responses), that is, the target sound detection threshold (BMTT). The obtained results established the ISI in which the masker began to interfere in pure tone perception in normal adults, which was considered the normative threshold of the test.

### 2.2. Procedures

After providing written informed consent and answering a questionnaire on their health, hearing, and educational history, all of the volunteers were submitted to the following tests:

(a) cognitive screening using the Brazilian version of the Montreal Cognitive Assessment (MoCA) protocol, with a score greater than or equal to 25 established as an inclusion criterion [41,42];
(b) basic hearing assessment (pure-tone audiometry, tympanometry and acoustic reflexes measurement), with a type A tympanogram, the presence of ipsi- and contralateral acoustic reflexes and hearing thresholds between 250 and 8000 Hz below 20 dBHL as inclusion criteria;
(c) behavioral assessment of ATP, using tests well established in clinical practice: the pitch pattern (PP; [43]), duration pattern (DP; [43]) and Gaps-in-Noise (GIN; [3]) tests, which assess temporal ordering and resolution. The tests were applied at an intensity of 50 dBSL, that is, 50 dB above the speech recognition threshold (SRT) of each individual;
(d) the backward masking test (BMT) developed in the present study, as specified in the previous section.

The interview and MoCA (item a) were applied in a quiet room, whereas items b, c, and d were carried out with participants seated in a sound booth, using a Grason-Stadler GSI-61 audiometer and TDH-50 headset. The prerecorded tests (items c and d) were applied to both ears separately, using a digital file player (Tablet) coupled to the audiometer. It is important to note that the first ear tested was alternated for each new participant to ensure that any performance differences per ear were not due to familiarization with the test or tiredness. 

### 2.3. Sample Characterization

For the standardization phase of the test, 43 adults aged between 18 and 40 years with no hearing or neurological deficits agreed to participate in the study. These individuals were family members or acquaintances of the researchers and/or employees and students at the university. They were invited to take part via flyers posted on bulletin boards, word of mouth, and social media posts. Twelve of these volunteers showed abnormal performance in both ears on at least one of the temporal processing tests applied (per item c above), or in at least one ear in more than two tests. Thus, only the data of 31 individuals (22 women) with an average age of 25.5 ± 5.9 years were used in this initial stage (NA group).

The data used in the second stage of the study, in addition to that of the NA group, were those of 12 young adults with abnormal ATP test results (AA group) and average age of 29.8 ± 6.2 years, and 17 older adults aged between 60 and 79 years (mean = 63.8 ± 4.9 years) (OA group). Besides an ATP deficit, all subjects in the AA group reported having hearing difficulties in tasks such as hearing in noise, following verbal instructions, and localizing sounds.

### 2.4. Results Analysis 

The data were submitted to descriptive analysis by calculating measures of central tendency and dispersion. In order to investigate the presence of interactions between the effects of SNR, ear and ISI duration on performance in the BMT in the NA group, a generalized estimated equation (GEE) model considering a binomial negative distribution for the dependent variable (reverse-transformed number of hits), was built. To verify the effect of ear and SNRs on the BMTT and BMTP measures in the NA group, GEEs with inverse Gaussian distributions for the dependent variables (BMTT and reverse-transformed BMTP) were built. BMT measures were compared between the three study groups by generalized linear models (GzLM) considering a gamma distribution for BMTT and a normal distribution for BMTP, with post hoc analysis using *t*-tests with sequential Bonferroni correction for multiple comparisons. For the GEEs, independent covariance matrix structures were considered for the GEEs and log link functions were used for all generalized models. All analyses were conducted using generalized models due to their flexibility in dealing with data with non-normal and/or heteroscedastic distribution [44,45].

Efficacy indices in the diagnosis of central hearing disorders using the BMT were calculated based on the “mean minus/plus 2 standard deviations” method, recommended by the main clinical guidelines in auditory processing disorder [46,47,48,49,50,51,52], and on the 10/90th percentile method, which can also be used for standardization of central auditory tests [43,53,54]. Both methods of calculation of cutoff points encountered in the literature were chosen so as to verify which of them yielded the best diagnostic indices for the BMT. Finally, correlations between the BMT and other ATP tests were investigated via Pearson’s correlation test. A 5% significance level was adopted for all of the inferential analyses. The effect sizes observed in the analyses were classified according to Cohen [55].

## 3. Results

**Figure 2:**
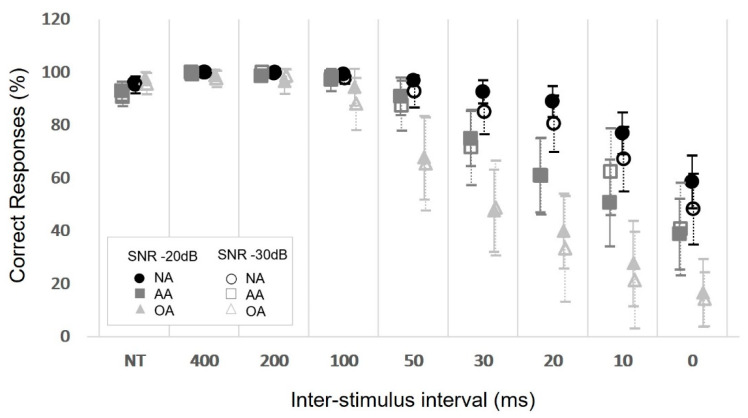
Distribution of the average number of correct responses per interstimulus interval for both SNRs in both ears for the three groups. SNR: signal-to-noise ratio; NA: normal adults group; AA: abnormal adults group; OA: older adults group; NT: no-tone. Error bar = 95% confidence interval.

Overall performance per ISI in the NA group remained constant at longer intervals (100–400 ms) and declined as the intervals shortened, from 50 ms onwards, in both ears and at both SNRs. In the AA group, performance started to decline at 100 ms, and, in the OA group, the decline in performance could be seen as early as 200 ms. The difference between groups’ performances per ISI became more pronounced as the ISIs shortened (Figure 2). For the NA group, there were no interactions between SNR, ear and ISI duration (SNR × ear × ISI: χ^2^ (7) = 11.414, *p* = 0.122; ear × ISI: χ^2^ (7) = 5.869, *p* = 0.555; SNR × ISI: χ^2^ (7) = 9.238, *p* = 0.236; SNR × ear: χ^2^ (1) = 0.166, *p* = 0.684). However, differences were observed in the number of correct responses between ISIs, regardless of the SNR or ear assessed (χ^2^ (7) = 212.532, *p* < 0.001), and between SNRs, regardless of the ISI or ear assessed (χ^2^ (1) = 4.697, *p* = 0.030). In general, for the ISIs, there was a statistically significant difference (*p* < 0.05) between the longest (100–400) and shortest time intervals (20, 10 and 0 ms), with significantly worse performance in the latter. For the SNR, worse performance was observed at −30 dB in comparison to the −20 dB condition. No statistically significant effect was observed for the ear assessed (χ^2^ (1) = 0.912, *p* = 0.340).

Regarding BMTP and BMTT in the NA group, no significant effect of ear was observed for both measures (BMTP: χ^2^ (1) = 0.005, *p* = 0.945; BMTT: χ^2^ (1) = 0.001, *p* = 0.978), and a significant effect of SNR was observed for BMTT (χ^2^ (1) = 4.055, *p* = 0.044), but not for BMTP (χ^2^ (1) = 2.651, *p* = 0.104) (Table 1). Considering the general absence of an ear effect in the analyses so far, both ears were grouped into a single sample for all of the study groups.

**Figure 3:**
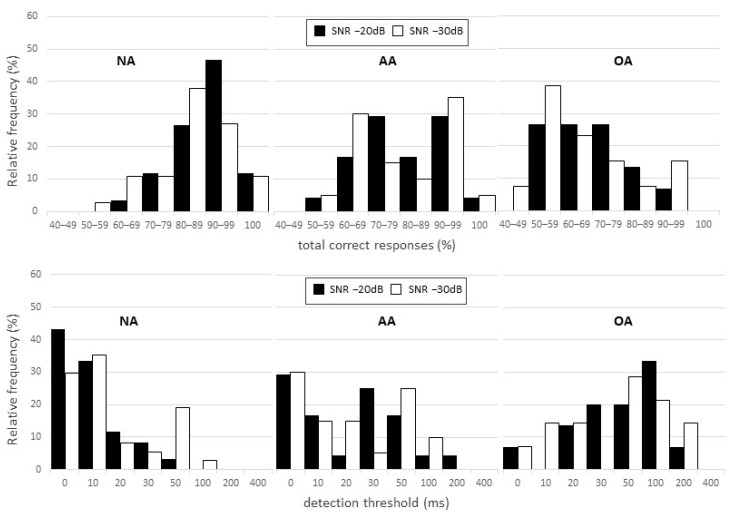
Distribution of the ears according to the percentage of correct responses (BMTP) and detection thresholds (BMTT) obtained in the backward masking test (BMT) for each signal-to-noise ratio (SNR). NA: normal adults group; AA: abnormal adults group; OA: older adults group.

Based on the distribution of participants according to test performance percentage, there was a slight shift in the distribution of results towards worse performance at SNR −30 dB for both BMTP and BMTT. However, performance in correct responses for most of the individuals in the NA group was equal to or greater than 80% at both ratios (SNR −20 dB: 85% of ears; SNR −30 dB: 82% of ears). Thresholds lower than or equal to 20 ms were recorded in 88% of ears at SNR −20 dB and 80% of ears at SNR −30 dB. Additionally, thresholds equal to or higher than 50 ms were obtained by only 3 and 14% of ears at SNRs of −20 and −30 dB, respectively. For the OA and AA groups, greater BMTP and BMTT variability was observed. In the AA group, 50% of ears had a BMTP of 80% or greater at both SNRs, while only 29 and 24% of ears in the OA group had a BMTP of 80% or greater at SNR −20 and −30 dB, respectively. Regarding BMTT, the AA group showed thresholds lower than or equal to 20 ms in 50% of ears at SNR −20 dB and in 60% of ears at SNR −30 dB, while thresholds equal to or higher than 50 ms were observed in 25 and 35% of ears in the respective conditions. For the OA group, these numbers were 29 and 35% of ears for thresholds lower than or equal to 20 ms and 53 and 65% of ears for thresholds equal to or higher than 50 ms (Figure 3). 

Significant differences were observed between the groups regarding the mean values of the BMT measures (BMTP at SNR -20 dB: χ^2^ (2) = 35.485; BMTT at SNR −20 dB: χ^2^ (2) = 34.466; BMTP at SNR −30 dB: χ^2^ (2) = 33.956; BMTT at SNR −30 dB: χ^2^ (2) = 33.573, all *p* values < 0.001). In general, the average performance of the AA and OA groups was worse than that of the NA group. Additionally, the OA group performed worse than the AA group in the BMT test, except for BMTT at SNR −20 dB (Table 2).

Based on data of the NA group, the values two standard deviations below or above the mean and at the 10 or 90th percentile were calculated to determine the best cutoff points for the test. 

Considering mean minus two standard deviations, the cutoff points for BMTP were 70.81% for the SNR −20 dB and 65.80% for the SNR −30 dB, which approximately correspond to 43/60 (71.67%) and 40/60 (66.67%) correct responses, respectively. Regarding BMTT, the cutoff points using the mean plus two standard deviations method were 33.83 ms for the SNR −20 dB and 55.95 ms for the SNR −30 dB, corresponding approximately to the possible BMTTs of 30 and 50 ms, respectively.

By the 10/90th percentile method, the cutoff points for BMTP were 75.00 and 68.33% (45 and 41/60 correct responses) for the SNR −20 and −30 dB, respectively. For BMTT, the cutoff points were 30 and 50 ms.

Based on the diagnostic indices for the suggested cutoff points, the BMT was more efficient at differentiating between the NA and OA than the NA and AA groups. Moreover, SNR −30 dB was less efficient than SNR −20 dB in both comparisons. Differences between the two measures and between the two methods were minimal, with a slightly better performance of BMTP over BMTT and of two standards below the mean over 10/90th percentile (Table 3). 

### Correlation with Other ATP Tests

Finally, a correlation analysis was performed with other ATP tests in a group containing all of the individuals assessed in this study (N = 60). There were low to moderate correlations between BMT and PPS and DPS test measures, as well as moderate to strong correlations between BMT and GIN test measures, indicating a significant degree of association between the performances of individuals in ATP tests (Table 4). 

## 4. Discussion

The BMT was developed to assess the ability of the central auditory nervous system (CANS) to mitigate the detrimental effect of BM on signal detection by changing the interval between the target sound and masker rather than altering intensity. This effect is clearly visible in Figure 2, where, from an ISI of 50 ms onwards, performance declines and higher variability is observed. This result is in accordance with the literature, which reports that BM has a greater effect for intervals up to approximately 25 ms, when it begins to decline until almost null at around 100 ms [4]. The lack of differences between longer and intermediate intervals and the significantly worse performance at shorter intervals, with increasingly larger differences between them, indicates that this characteristic of BM is present in the BMT. Moreover, the more detrimental effect of BM observed in the BMT in groups that can be considered as having a less efficient CANS, such as older adults and those with abnormal performance on well-established ATP tests, reinforces evidence that the test proposed in this study is a valid measure of BM. Correlations were observed with other ATP tests, demonstrating that the BMT does in fact assess an ATP phenomenon and further substantiating the construct validity of the test.

The BMT demonstrated worse diagnostic performance at the more difficult SNR, as observed in detection threshold analysis. There are reports in the literature that intensity has a far greater effect for forward masking than BM [56,57]. As such, the difference observed in the BMT can be explained by the fact that the procedure used to modify the SNR was to reduce tone intensity rather than increase noise intensity. Thus, at SNR −30 dB, the tone was presented at an intensity close to the hearing threshold of the individual for that tone, which may have resulted in greater uncertainty/indecision on the part of some participants, increasing the involvement of attention in executing the task. This hypothesis is supported by the greater variability observed in the groups at SNR −30 dB and participant distribution according to performance (Figure 3). Other studies [20,58] have reported the influence of attention in other auditory processing tests, including ATP tests, and indicate the need for these instruments to be able to differentiate it from central auditory function in order to obtain more accurate audiological diagnoses.

There were no differences between ears for the BMT in the present study. Thus, in order to shorten the application time of a battery of tests to assess CAPD, it is suggested that only one ear be evaluated. The BMT is already a rapid test. With a track duration of around 5 min, application of two test tracks and a training track means total duration is approximately 15 min. By applying the test to only one ear, it can be completed in only 10 min. 

Around 80% of the ears of the NA group showed a percent correct of 80% or higher and detection threshold of 20 ms or shorter, with a greater concentration of ears in the 80–90% and 0 to 10 ms ranges. Although the detection threshold seems to suffer a ceiling effect, with around 40% of ears with a detection threshold of 0 ms, this effect seems to be much less pronounced in the percent correct, with around 10% of ears with a correct percentage of 100%. The absence of a ceiling effect is a desirable feature in a diagnostic test [59] and, regarding this point, the percent correct may be a less biased measure for the BMT.

The NA group performed significantly better and showed less variability than the AA and OA groups. Performance in the OA group was significantly worse at both SNRs, unlike AA participants, who did not exhibit a statistical difference in BMTT at SNR −30 dB. This raises two important points. First, a lower SNR should make it more difficult for the central nervous system to recover from the masking effect, especially in individuals characterized as having an auditory processing disorder. The similar performance among the young adults at SNR −30 dB may be an indication that factors other than ATP played an important role in performance. As previously mentioned, uncertainty and attention may have been more important at SNR −30 dB than SNR −20 dB. These observations, combined with the fact that the diagnostic analyses demonstrated worse performance for the test at SNR −30 dB, suggest that it should be applied at SNR −20 dB. Regarding the use of BMTT and BMTP, although BMTP had a better diagnostic accuracy, this difference was minimal, and future studies should investigate which measure best represents an individual’s ATP skills.

A second point regarding performance among the groups is that the older adults exhibited a statistically significant difference in relation to both groups of young adults, as observed in other studies that assessed the detrimental effect of BM in this age range [30]. Research has demonstrated that aging has a negative effect on information processing as a whole [34,35]. The clear difference between young and older adults might be an indication of this decline in general processing and not only in ATP. Specific standardization for older adults is suggested in order to evaluate the role of auditory processing in the performance of this population for BM. 

The absence of a gold standard population for auditory processing disorders (neurological changes associated with the temporal cortex) is a limitation of the present study. The standardization proposed here considered the performance of young adults with abnormal ATP and older adults, individuals with high heterogeneity who do not constitute clinical populations per se. A second limitation refers to the choice to group both ears of each group. Although this procedure might have compromised the p-values calculated in the present study due to a possible violation of the assumption of independence of observations, it may be an interesting move in order to reduce the number of measures to be analyzed in clinical practice and to increase statistical power of the sample. It is also worth noting that grouping the ears together is a data analysis procedure used in other studies in the audiology field (e.g., [60,61,62,63,64]). Another limitation might be the predominance of female subjects in our sample, although previous research found no difference between genders regarding BMT performance [31].

The cutoff values with the best diagnostic indices for the SNR −20 dB were 75.00% correct responses and 30 milliseconds, both calculated through the 10/90th percentile method. At these values, 59 and 38% of ears among the older adults and those with abnormal ATP, respectively, showed abnormal performance for the total percentage of correct answers, while only 7% of normal individuals performed abnormally. In analysis based on the target sound detection threshold, abnormal performance was observed in 53 and 25% of ears among the group of older adults and those with ATP alterations, respectively, and only 3% in the NA group. Although sensitivity values (i.e., true-positive rate) are considerably low, probably due to the lack of a more adequate abnormal population (e.g., individuals with neurological disorders), specificity values (i.e., true-negative rate) are very good [65]. In other words, a “normal” individual has a high probability of performing within normal limits in the BMT, while an abnormal performance is a strong indicator of abnormal CANS function. 

It is important to underscore that these diagnostic indices were obtained only for the BMT. Since auditory processing disorders are evaluated in a battery of tests, diagnosis depends on the performance of the individual in different tests. Future studies should be performed to determine the diagnostic indices of a battery of tests that includes the BMT, preferably using individuals with neurological lesions. The correlations between the BMT and other ATP tests indicate that its association with other tests tends to enhance its diagnostic value [66]. Additionally, another relevant part of the validation process of a new clinical tool is the evaluation of test-retest reliability, which should be addressed in future studies using the BMT. Finally, new studies should investigate other populations’ performance in the BMT, such as children, musicians, and individuals with language or speech disorders.

## 5. Conclusions

This study produced a test to assess the effect of BM, an essential part of the auditory processing of speech. The results of the test support the theory regarding BM. Moreover, the test is quick and easy to apply and can be used in only one ear with an SNR of −20 dB. The normative values suggested were 75.00% for performance based on percentage of correct responses and 30 milliseconds for the detection threshold. Based on the proposed cutoff values, the BMT was shown to have considerable potential in demonstrating adequate ATP in normal individuals, while an abnormal result is a strong indicator of ATP disorder.

## Figures and Tables

**Table 1:** Descriptive statistics for performance in percentage of correct responses (BMTP) and target sound detection threshold (BMTT) in the NA group for the backward masking test (BMT), according to the signal-to-noise ratio (SNR) and ear assessed.

	SNR (dB)	Ear	Mean	SD	Minimum	Median	Maximum
BMTP	−20	Right	88.70	9.40	63.00	90.00	100
		Left	89.66	9.04	65.00	93.33	100
	−30	Right	87.85	10.34	55.00	88.33	100
		Left	85.86	11.39	61.67	87.50	100
BMTT	−20	Right	10.65	13.89	0	10	50
		Left	8.97	9.76	0	10	30
	−30	Right	13.55	20.58	0	10	100
		Left	18.33	20.07	0	10	50

BMTP: Percentage of correct responses in the BMT; BMTT: detection threshold in the BMT; SD: standard deviation.

**Table 2:** Descriptive statistics and comparison of performance in percentage of correct responses (BMTP) and target sound detection threshold (BMTT) for the three groups for the backward masking test (BMT), according to the signal-to-noise ratio (SNR).

	SNR (dB)		Mean	SD	Minimum	Median	Maximum	*p* Value	
								**AA/OA vs. NA**	**OA vs. AA**
BMTP	−20	NA	89.16	9.16	63.00	90.80	100	--	--
		AA	80.61	13.33	55.00	80.82	100	0.003 *	0.006 *
		OA	70.98	14.22	51.66	70.00	96.66	<0.001 *	
	−30	NA	87.12	10.66	55.00	88.33	100	--	--
		AA	80.23	14.81	56.66	81.66	100	0.040 *	0.002 *
		OA	66.47	16.01	45.00	61.66	93.33	<0.001 *	
BMTT	−20	NA	9.83	12.00	0	10	50	--	--
		AA	30.83	43.43	0	25	200	0.014 *	0.138
		OA	58.24	52.11	0	50	200	0.013 *	
	−30	NA	15.31	20.32	0	10	100	--	--
		AA	28.50	31.33	0	20	100	0.058	0.001 *
		OA	65.88	61.14	0	50	200	<0.001 *	

*: statistically significant difference (*p* < 0.05) BMTP: percentage of correct responses in the BMT; BMTT: detection threshold in the BMT; SD: standard deviation; vs.: versus; SNR: signal-to-noise ratio; NA: normal adults group; AA: abnormal adults group; OA: older adults group.

**Table 3:** Indices (%) of specificity, sensitivity, efficiency, positive, and negative predictive values of the backward masking test (BMT) for each testing condition, method of calculation, and comparison group.

	SNR (dB)	Method	Specificity (%)	Sensitivity (%)		PPV (%)		NPV (%)		Efficiency (%)	
				AA	OA	AA	OA	AA	OA	AA	OA
BMTP	−20	M − 2SD	95	29	59	85	92	57	70	62	77
		P	93	38	59	84	89	60	69	66	76
	−30	M − 2SD	94	30	59	83	91	57	70	62	76
		P	92	30	59	79	88	57	69	61	75
BMTT	−20	M + 2SD	97	25	53	88	94	56	67	61	75
		P	97	25	53	88	94	56	67	61	75
	−30	M + 2SD	98	10	35	83	95	52	60	54	67
		P	98	10	35	83	95	52	60	54	67

PPV = Positive predictive value; NPV = Negative predictive value; BMTP: percentage of correct responses in the BMT; BMTT: detection threshold in the BMT; SNR: signal-to-noise ratio; M − 2SD: mean minus two standard deviations; M + 2SD: mean plus two standard deviations; P: percentile; AA: abnormal adults group; OA: older adults group.

**Table 4:** Correlation coefficients between backward masking test (BMT) measures and performance in clinical auditory temporal processing tests for the total sample.

		GP	GT	PP	DP
BMTP (SNR −20 dB)	Coeff.	0.572	−0.545	0.289	0.355
	*p*	<0.001 *	<0.001 *	0.008 *	<0.001 *
BMTT (SNR −20 dB)	Coeff.	−0.549	0.486	−0.366	−0.211
	*p*	<0.001 *	<0.001 *	<0.001 *	0.039 *
BMTP (SNR −30 dB)	Coeff.	0.549	−0.510	0.421	0.419
	*p*	<0.001 *	<0.001 *	<0.001 *	<0.001 *
BMTT (SNR −30 dB)	Coeff.	−0.508	0.500	−0.403	−0.286
	*p*	<0.001 *	<0.001 *	<0.001 *	0.009 *

Coeff.: coefficient; *: *p* < 0.05; BMTP: percentage of correct responses in the BMT; BMTT: detection threshold in the BMT; SNR: signal-to-noise ratio; GP: Gaps-in-Noise test percentage; GT: Gaps-in-Noise test threshold; PP: pitch pattern test percentage; DP: duration pattern test percentage. Correlation analyses were carried out with both ears grouped into a single sample.

## Data Availability

The data presented in this study are available on request from the corresponding author. The data are not publicly available due to ethical issues.

## Supplementary Materials

The following supporting information can be downloaded at: https://www.mdpi.com/article/10.3390/jcm11174933/s1, Annex S1: Backward Masking Test answer sheet.
