# Supplementary material for: Evidence of Validity and Normative Values of a New Auditory Backward Masking Test
Source: J Clin Med. 2022 Aug 23;11(17):4933. doi: 10.3390/jcm11174933 (PMC9456366; doi:10.3390/jcm11174933)
Supplement: Supplementary file 1 [file jcm-11-04933-s001.zip › jcm-1813916-supplementary.pdf]

Annex S1: Backward Masking Test answer sheet.

AUDITORY TEMPORAL MASKING TEST (FILIPPINI, R)

PRACTICE LIST

	FIRST	SECOND
**1**	400	400
**2**	400	200
**3**	--	--
**4**	400	100
**5**	400	--
**6**	--	400
**7**	400	50
**8**	400	100
**9**	--	200
**10**	400	--
**11**	400	50
**12**	--	100
**13**	400	30
**14**	400	200
**15**	--	400

NAME/ID

DATE OF BIRTH *DATE OF ASSESSMENT*

OBS.:

LIST 1

EAR: ________

**1**	400	**31**	100
**2**	100	**32**	20
**3**	30	**33**	--
**4**	50	**34**	10
**5**	--	**35**	50
**6**	0	**36**	30
**7**	20	**37**	100
**8**	--	**38**	30
**9**	200	**39**	--
**10**	10	**40**	0
**11**	--	**41**	200
**12**	30	**42**	--
**13**	50	**43**	10
**14**	0	**44**	20
**15**	--	**45**	100
**16**	20	**46**	200
**17**	100	**47**	50
**18**	400	**48**	400
**19**	0	**49**	--
**20**	10	**50**	10
**21**	100	**51**	0
**22**	200	**52**	400
**23**	10	**53**	--
**24**	--	**54**	30
**25**	400	**55**	50
**26**	20	**56**	400
**27**	50	**57**	20
**28**	--	**58**	0
**29**	30	**59**	--
**30**	200	**60**	200

LIST 2

EAR: ________

**1**	400	**31**	20
**2**	100	**32**	--
**3**	200	**33**	100
**4**	--	**34**	50
**5**	30	**35**	--
**6**	50	**36**	0
**7**	10	**37**	400
**8**	100	**38**	30
**9**	20	**39**	200
**10**	--	**40**	10
**11**	0	**41**	200
**12**	400	**42**	20
**13**	--	**43**	400
**14**	30	**44**	--
**15**	10	**45**	50
**16**	200	**46**	100
**17**	10	**47**	30
**18**	0	**48**	--
**19**	--	**49**	30
**20**	30	**50**	--
**21**	200	**51**	0
**22**	50	**52**	--
**23**	--	**53**	400
**24**	50	**54**	0
**25**	400	**55**	10
**26**	20	**56**	50
**27**	0	**57**	20
**28**	20	**58**	100
**29**	100	**59**	200
**30**	--	**60**	10

RESULTS

LIST 1

TONE THRESHOLD: ______

TONE INTENSITY: ______

MASK INTENSITY: ______

INTERVAL THRESHOLD

400 - .... /6
200 - .... /6
100 - .... /6
50 - .... /6
30 - .... /6
20 - .... /6
10 - .... /6
0 - .... /6

LIST 2

TONE THRESHOLD: ______

TONE INTENSITY: ______

MASK INTENSITY: ______

INTERVAL THRESHOLD

400 - .... /6
200 - .... /6
100 - .... /6
50 - .... /6
30 - .... /6
20 - .... /6
10 - .... /6
0 - .... /6
